# Transition Metal Oxodiperoxo Complex Modified Metal-Organic Frameworks as Catalysts for the Selective Oxidation of Cyclohexane

**DOI:** 10.3390/ma13040829

**Published:** 2020-02-12

**Authors:** Yuechao Hong, Jie Peng, Zhichao Sun, Zhiquan Yu, Anjie Wang, Yao Wang, Ying-Ya Liu, Fen Xu, Li-Xian Sun

**Affiliations:** 1 State Key Laboratory of Fine Chemicals, School of Chemical Engineering, Dalian University of Technology, Dalian 116024, China; h15541172820@163.com (Y.H.); peng.jie.p4@dc.tohoku.ac.jp (J.P.); sunzhichao@dlut.edu.cn (Z.S.); yuzhiquan@dlut.edu.cn (Z.Y.); ajwang@dlut.edu.cn (A.W.); wangyao@dlut.edu.cn (Y.W.); 2 Guangxi Key Laboratory of Information Materials and Guangxi Collaborative Innovation Center of Structure and Property for New Energy and Materials, School of Material Science & Engineering, Guilin University of Electronic Technology, Guilin 541004, China; xufen@guet.edu.cn (F.X.); sunlx@guet.edu.cn (L.-X.S.)

**Keywords:** metal-organic framework, cyclohexane oxidation, transition metal oxodiperoxo complexes, cyclohexanol, cyclohexanone

## Abstract

In this work, a series of modified metal-organic frameworks (MOFs) have been prepared by pre- and post-treatment with transition metal oxodiperoxo complexes (MoO(O_2_)_2_, WO(O_2_)_2_, and KVO(O_2_)_2_). The obtained materials are characterized by XRD, FTIR, SEM, TEM, inductively coupled plasma atomic emission spectrometry (ICP-AES), and X-ray photoelectron spectroscopy (XPS), as well as by N_2_ adsorption/desorption measurement. The characterization results show that transition metal oxodiperoxo complexes are uniformly incorporated into the MOF materials without changing the basic structures. The performance of cyclohexane oxidation on metal oxodiperoxo complex modified MOFs are evaluated. UiO-67-KVO(O_2_)_2_ shows the best performance for cyclohexane oxidation, with 78% selectivity to KA oil (KA oil refers to a cyclohexanol and cyclohexanone mixture) at 9.4% conversion. The KA selectivity is found to depend on reaction time, while hot-filtration experiments indicates that the catalytic process is heterogeneous with no leaching of metal species.

## 1. Introduction

Selective C-H oxidation is of crucial importance in activating raw materials to produce oxygenates in many industrial processes [1,2]. The oxidation of cyclohexane is the main industrial process to obtain a mixture of cyclohexanone (Cyone) and cyclohexanol (CyOH) (referred to as KA oil of ketone and alcohol mixture), which are important intermediates in the production of Nylon-6 and Nylon-66 [3,4,5], and are also raw materials for the production of caprolactam and adipic acid [6]. In the oxidation process, the deep oxidation often results in the formation of undesired by-products [7]. In the industrial process, reasonable selectivity to the desired intermediate KA oil is achieved at a lower conversion [8,9]. Therefore, the selective oxidation of cyclohexane at mild condition with high energy efficiency is one of the major needs and biggest challenges for sustainable industry [10,11]. To tackle this issue, there is an urgent need for efficient, high selectivity, environmentally benign catalysts towards the sustainable development of such an important oxidation process [12]. Primarily, there are two commercial processes in cyclohexane oxidation: The non-catalytic autoxidation process and the catalyzed process [13]. In both processes, radical chemistry plays a major role. Many transition metals (Ti, V, Cr, Co, Mn, Fe, Mo) in their oxide forms [14,15,16] are used as catalyst in cyclohexane oxidation reactions, and silica-based material is a common choice as the catalyst support [17,18,19,20,21]. For instance, a mesoporous siliceous material, TUD-1, has been functionalized by different metals, and the obtained M-TUD-1 (M = Co, Cr, Cu, Mn, Mo, Ti, and V) was examined in the liquid phase oxidation of cyclohexane with tert-butyl hydroperoxide (TBHP) as the oxidant. Co-TUD-1 has shown good catalytic activity in cyclohexane conversion, but the radical product, cyclohexyl hydroperoxide (CHHP), is produced in large amounts [22,23]. In a recent study, by introducing a second metal (Mn, Ti, V, or Bi) into Co-TUD-1, the bimetallic TUD-1 showed higher activity and less selectivity towards CHHP in the oxidation of cyclohexane [21]. During the study, metal leaching was one of the main problems in the liquid phase oxidation of cyclohexane because the impregnation, ion-exchange, or isomorphous substitution cannot form strong bonding between the metal species and the support [24,25,26].

**Scheme 1:**
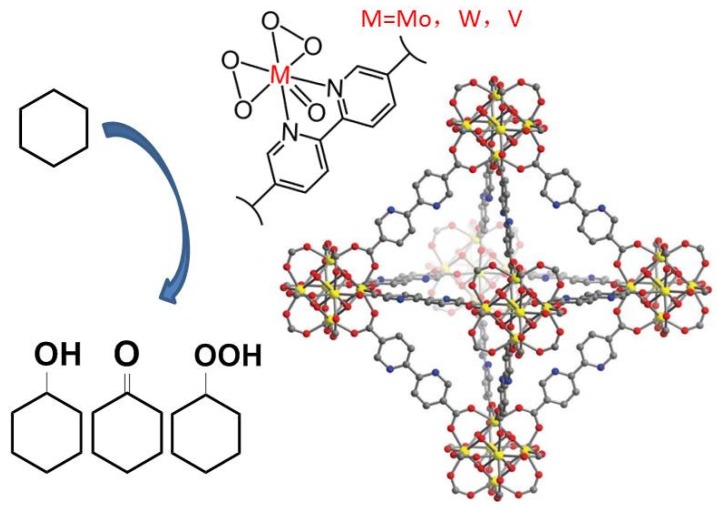
Illustration of modified UiO-67 as a catalyst for the selective oxidation of cyclohexane.

Metal-organic frameworks (MOFs) have received extensive attention in the field of heterogeneous catalysis due to their high porosity, abundant pore structure, large specific surface area, and easy modification [27,28,29]. MOFs are crystalline materials constructed by linking multidentate ligands with metal/cluster nodes to form infinite networks. The strong coordination interactions between metal and ligand and the infinite three-dimensional structures ensure the good chemical stability of the MOFs and allow for rational design and synthesis [30]. 2,2′-Bipyridine (bpy) is one of the most widely used bidentate chelators in transition metal coordination chemistry [31]. It is very interesting and useful for combining the excellent hydrocarbon oxidation properties of transition metals with the superior properties of MOFs. In our previous work, a molybdenum complex, MoO_2_Cl_2_, was tethered to the bipyridine linkers of a Ga-MOF (COMOC-4), where the catalyst showed high performance in the liquid phase oxidation reactions, such as epoxidation of cycloalkenes and oxidation of polyaromatic sulfur compounds [32,33]. Lin’s group also worked on MOFs of UiO topology containing a bipyridine ligand by anchoring Ir complexes onto the MOFs, and their catalyst showed superior activity and reusability in C−H activation [34]. Most recently, by loading polyoxovanadates (POVs) onto MOFs, mixed-valence {V16} [35] cluster-based MOF catalysts were prepared, which showed remarkable catalytic performance (24.6% conversion with 99% selectivity to KA oil) in the aerobic oxidation of cyclohexane. The results also showed that the mixed-valence {V16} cluster exhibited higher activity than the homovalent vanadium cluster. In this work, a metal-organic framework, UiO-67, with linker replaced by 2,2′-bipyridine-5,5′-dicarboxylate (UiO-67-bpydc) (Scheme 1) was used as the solid support. Zr-based MOFs are known for their exceptional thermal, chemical, and mechanical stability properties, which are important for catalytic applications [36]. Among them was a Universuty of Oslo (UiO) series of Zr-based MOFs comprising 12-connected Zr_6_-oxide nodes, featuring an **fcu** topology. The pore aperture size of UiO-67 is between 8 and 11.5 Å, allowing further functionalization [37,38]. The original UiO-67 was reported with biphenol dicarboxylate ligand; with 2,2′-bipyridine-5,5′-dicarboxylate as the replacing ligand, it is easy to introduce metal active components onto the bipyridine nitrogen for functionalization due to its own chelation sites. Herein, a series of active components, peroxo vanadates (pV), peroxo compounds of molybdenum (pMo), and tungsten (pW), were anchored via 2,2′-bipyridine sites. The catalytic activity of the obtained materials, namely UiO-67-MO(O_2_)_2_ (M = Mo, W, V), were evaluated in the liquid phase oxidation of cyclohexane.

## 2. Material and Methods

### 2.1. Material

2,2′-bipyridine-5,5′-dicarboxylic acid (H_2_bpydc) was prepared following a published method [39]. MoO(O_2_)_2_·2DMF (DMF = *N*, *N*-dimethylformamide) was prepared according to the published procedure [40]. All other chemicals were obtained commercially and used without further purification.

The powder X-ray diffraction (XRD) patterns were recorded on a Rigaku D-Max-2400 diffractometer using Cu Kα radiation at 40 kV and 100 mA. The metal content in the samples was determined by inductively coupled plasma atomic emission spectrometry (ICP-AES) (Optima 2000DV). Fourier transform infrared spectra (FTIR) was recorded on an FT-IR-800 spectrometer in KBr disks at room temperature. The specific surface area of the catalyst was determined by N_2_ adsorption/desorption isotherm using a Tristar II 3020 adsorption analyzer (The data are treated according to the Brunauer, Emmett and Teller (BET) adsorption isotherm equation). Before the measurement, the sample was degassed at 120 °C under vacuum overnight. The morphology and structures of the catalysts were characterized using scanning electron microscopy (SEM) (NOVA nanoSEM 450). X-ray photoelectron spectroscopy (XPS) measurements were recorded on a Multilab 2000 X-ray photoelectron spectrometer using an Al Kα source. The binding energies of all the observed peaks were calibrated towards a value for the C 1 s peak of adventitious carbon at 284.6 eV.

### 2.2. Catalyst Preparation

#### 2.2.1. Synthesis of UiO-67-bpydc

UiO-67-bpydc (**1**) was synthesized according to a reported procedure with slight modification [31]. A mixture of ZrCl_4_ (0.245 g, 1.05 mmol), H_2_bpydc (0.256 g, 1.05 mmol), acetic acid (HOAc) (1.8 mL), and DMF (50 mL) was added in a Schlenk flask and sonicated for 20 min, then sealed and held at 120 °C for 24 h under stirring. After cooling down to room temperature, the precipitate was isolated by centrifugation. The powder product was suspended in DMF (50 mL) at 80 °C for 3 h under stirring and isolated by centrifugation. The obtained white powder was washed by ethanol twice and dried at 100 °C overnight (yield: 89% based on Zr).

#### 2.2.2. Synthesis of UiO-67-MoO(O_2_)_2_

UiO-67-MoO(O_2_)_2_ (**2**) was prepared by post-synthetic modification following a reported procedure [40]. Typically, MoO(O_2_)_2_·2DMF (0.36 g, 1.12 mmol) was fully dissolved in ethanol (80 mL) under vigorous stirring, and **1** (0.4 g, 0.187 mmol) was added into the solution and stirred vigorously at 80 °C for 24 h. The solution was cooled to room temperature and the precipitate was isolated by centrifugation. The solid product was washed by ethanol twice and dried at 100 °C overnight (yield: 85% based on UiO-67).

#### 2.2.3. Synthesis of UiO-67-WO(O_2_)_2_

UiO-67-WO(O_2_)_2_ (**3**) was synthesized by preparing the partially metalated ligand and then building the MOF structure. Peroxotungstate solution was prepared following a reported procedure with modification [41]. A mixture of H_2_WO_4_ (4.5 g, 18 mmol) and H_2_O_2_ (40 mL, 30% by weight solution) was stirred at 45 °C for 24 h. Then the solution was centrifuged and the supernatant liquid was placed in the refrigerator (2 °C–4 °C) prior to use. H_2_bpydc (1 g, 4.1 mmol) was added into the supernatant (20 mL) and then stirred at room temperature for 24 h. The solution was centrifuged, and the solid was washed by ethanol twice. After drying at 100 °C overnight, H_2_bpydc-WO(O_2_)_2_ precursor was obtained with a yield of 0.98 g.

A mixture of ZrCl_4_ (0.245 g, 1.05 mmol), H_2_bpydc-WO(O_2_)_2_ (0.534 g), HOAc (1.8 mL), and DMF (50 mL) was added in a Schlenk flask and sonicated for 20 min, then sealed and stirred at 120 °C for 24 h. The solution was cooled to room temperature, and the precipitate was isolated by centrifugation. The solid product was washed by DMF once, followed by ethanol twice and finally dried at 100 °C overnight. (yield: 82% based on Zr).

#### 2.2.4. Synthesis of UiO-67-KVO(O_2_)_2_

UiO-67-KVO(O_2_)_2_ (**4**) was synthesized by preparing the partially metalated ligand and then building the MOF structure. Peroxovanadate precursor solution was prepared following the reported procedure with modification [42]. H_2_bpydc (0.2442 g, 1 mmol) was added into ethanol (20 mL) and suspended using ultrasound for 30 min to form a suspension (**a**). KVO_3_ (0.138 g, 1 mmol) was dissolved into H_2_O_2_ (10 mL) in a beaker to give a solution (**b**) by stirring in an ice water bath. All steps described subsequently in this paragraph were operated in an ice water bath. Solution **a** was added drop wise into solution **b**, with constant stirring. After the addition was completed, the yellow mixture was stirred for 5 min, then 10 mL of cold ethanol was added. After 10 min, the mixture was allowed to stand for 15 min for stratification. Then, another 10 mL of cold ethanol was added, and the mixture was stirred for 10 min and then left to stand for another 10 min. The solid product was isolated by centrifugation and washed by ethanol twice and finally dried at room temperature in vacuum. We then obtained the light yellow H_2_bpydc-KVO(O_2_)_2_ precursor with a yield of 0.25 g.

A mixture of the obtained solid (H_2_bpydc-KVO(O_2_)_2_, 0.171 g), ZrCl_4_ (0.082 g, 0.35 mmol), HOAc (0.6 mL), and DMF (17 mL) was added in a sealed glass flask and sonicated for 20 min; it was then stirred at 120 °C for 24 h. The solution was cooled to room temperature and the precipitate was isolated by centrifugation. The solid product was washed by DMF once and by ethanol twice and finally dried at 80 °C overnight. (yield: 88% based on Zr).

### 2.3. Catalytic Oxidation of Cyclohexane

The oxidation of cyclohexane was performed in a 35 mL Pyrex tube under stirring. In a typical reaction, the cyclohexane (8 mmol), tert-butyl hydroperoxide (TBHP, 8 mmol, 70 wt% in water), and 1,2,4-trichlorobenzene (2.78 mmol) (the internal standard) were added into 8 mL acetonitrile. UiO-67-MO(O_2_)_2_ (M = Mo, W, V) was added as a catalyst with a molar ratio of the second metal to cyclohexane of 1:266. After the reaction, the spent catalyst was separated by centrifugation and washed with acetone, dried at room temperature, and subsequently reused.

### 2.4. Product Analysis

The oxidation products include the target products CyOH and Cyone, a radical product, CHHP, and by-products such as organic acids and esters. In our catalytic reaction system, only organic acid was detected, and no ester product was found in the titration.

(1) CyOH and Cyone

Cyclohexane, CyOH, and Cyone were analyzed by gas chromatography (GC) with HP-5 capillary column (30 m × 0.32 mm). Quantitative analysis of cyclohexane, CyOH, and Cyone was performed by the internal standard curve method.

(2) Peroxide

The peroxides in the reaction solution included the oxidant TBHP and the intermediate product CHHP, which were quantified by an indirect iodometric method. The peroxide concentration was calculated as follows:

(1) $$C=\frac{\left(V-V_{0}\right)\times C_{0}}{2\times V_{\mathrm{sample}}}$$

where C is the peroxide concentration, mol/L; V is the consumption sodium thiosulfate (Na_2_S_2_O_3_) standard solution volume, mL; V_0_ is the blank experiment consumption Na_2_S_2_O_3_ standard solution volume, mL; C_0_ is the Na_2_S_2_O_3_ standard solution concentration, and mol/L; V_sample_ is the sample volume, mL.

(3) CHHP

The radical product, CHHP, is thermally unstable and will be partially decomposed into CyOH and Cyone in GC, which affects the analysis results. An excess of triphenylphosphine (PPh_3_) was added to the sample to completely reduce CHHP to CyOH, and the CHHP content was calculated by chromatography before and after PPh_3_ addition. The simplified formula is:

(2) $${\left[A\right]}_{\mathrm{PPh}3}=\left[A\right]+\left[\mathrm{CHHP}\right]$$

where [A]_PPh3_ is the result of chromatographic analysis of CyOH after the addition of PPh_3_; [A] is the result of chromatographic analysis of CyOH before the addition of PPh_3_; and [CHHP] is CHHP content.

## 3. Results and Discussion

### 3.1. Synthesis and Characterization

#### 3.1.1. Synthesis

UiO-67-MoO(O_2_)_2_ is prepared by treating a suspension of the pristine UiO-67 in ethanol with MoO(O_2_)_2_·2DMF. This post-synthetic approach was commonly used to provide MOFs with new functionalities by a solid-liquid reaction [43]. However, the attempt to prepare UiO-67-WO(O_2_)_2_ and UiO-67-KVO(O_2_)_2_ by post-synthetic modification was not successful because WO(O_2_)_2_ and KVO(O_2_)_2_ complexes can only form a stable solution in concentrated hydrogen peroxide solution, where the MOF structure cannot survive in such a harsh condition. Alternatively, UiO-67-WO(O_2_)_2_ and UiO-67-KVO(O_2_)_2_ are both prepared by coordinating first with the organic ligand and then by building the MOF structure. The WO(O_2_)_2_ and KVO(O_2_)_2_ anchored organic ligand were prepared by the standard synthetic method for complexes of the type [MO(O_2_)_2_L] (L = organic ligand), which involves adding the organic ligand (H_2_bpydc in our case) to the aqueous solution containing the [MO(O_2_)_2_ (M = W and V)] complex and a large excess of H_2_O_2_ [41,42]. Please note that the anchoring procedure of the organic ligand is incomplete; only part of the ligand was successfully functionalized. ICP and elemental analysis showed that the metalated linker has a doping molar ratio of 0.31 for W and 0.35 for V (Table S1, Supplementary Material), FTIR spectra of H_2_bpydc after metalation showed a band at 1684 cm^−1^, which was assigned to the –COOH stretching vibration (Figure S1, Supplementary Material). The characterization demonstrated that the free carboxylic acid group is still available for further coordination with Zr salt to build the MOF framework. However, such a mixed-ligand was sufficient enough to build the MOF framework containing certain amounts of oxodiperoxo complexes.

#### 3.1.2. XRD Characterization

**Figure 1:**
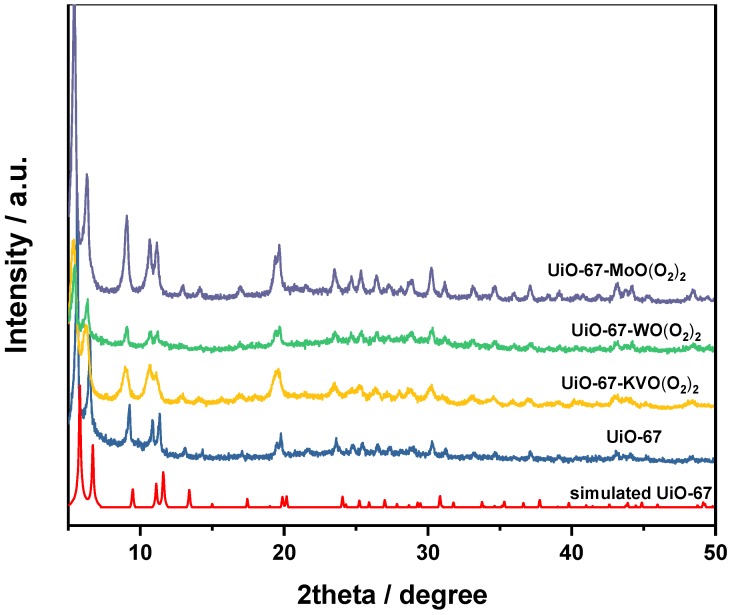
XRD patterns of the simulated UiO-67 and the synthesized UiO-67, UiO-67-MoO(O_2_)_2_, UiO-67-WO(O_2_)_2_, and UiO-67-KVO(O_2_)_2_.

In Figure 1, the simulated XRD pattern of UiO-67 derived from single-crystal x-ray diffraction data and the experimentally synthesized UiO-67 before and after modification are compared. The crystal structure of the experimentally synthesized MOF material was consistent with the simulated XRD pattern, indicating that the structural integrity was well preserved after modification. After anchoring MoO(O_2_)_2_, WO(O_2_)_2_, and KVO(O_2_)_2_, the position of the diffraction peak was unchanged, but the intensity was decreased, especially for UiO-67-KVO(O_2_)_2_. However, no additional diffraction peak appeared. It indicates that the MOF material had a decreased crystallinity after metal peroxide modification, but the framework structure was intact. The supported transition metal peroxide was highly dispersed on the MOF framework, and there was no characteristic diffraction of the transition metal oxide or metal complexes.

#### 3.1.3. Nitrogen Physical Adsorption, ICP-AES, and FTIR Studies

The metal loading of the modified MOF materials and BET specific surface areas are listed in Table 1. The loadings of Mo, W, and V were determined by ICP analysis and were 14.1 wt%, 6.3 wt%, and 2.7 wt%, respectively. It should be noted that, although the MoO(O_2_)_2_ was tethered on the framework by post-synthetic modification, the loading of the Mo complex was very high. There were 4.2 MoO(O_2_)_2_ molecules in one UiO-67 structural unit (Table 1). The WO(O_2_)_2_ and KVO(O_2_)_2_ complexes were anchored on the MOF frameworks by coordinating first with the organic ligand and then by building the MOF structure. There were certain losses of metal oxodiperoxo complexes during the formation of MOFs, as evidenced by ICP and elemental analysis. Metalated ligand has a metal to ligand molar ratio of 0.35 for V (Table S1, Supplementary Material), while UiO-67-KVO(O_2_)_2_ has a metal to MOF molar ratio of 1.2, which indicates that there is 57% of KVO(O_2_)_2_ remaining on the MOF framework. Similarly, 43% of WO(O_2_)_2_ was anchored onto the MOF framework compared to the partially metalated linker. The BET specific surface area of UiO-67 was 1562 m^2^/g, and after pre- and post-modification, the surface areas of UiO-67-MoO(O_2_)_2_, UiO-67-WO(O_2_)_2_, and UiO-67-KVO(O_2_)_2_ significantly reduced to 583 m^2^/g, 656 m^2^/g, and 305 m^2^/g, respectively, which may be due to the fact that the metal complexes can occupy and/or block the cavities of the UiO-67, and the MOF structure partially collapsed during the modification process. As shown in Figure S2 (Supplementary Material), UiO-67-MoO(O_2_)_2_ and UiO-67-KVO(O_2_)_2_, when prepared with different procedures activated by heating (100 °C), have both shown certain losses in crystallinity compared to room temperature activation. The decrease in surface area was in agreement with the XRD results, indicating that there was certain loss in crystallinity during every step of the modification process.

**Figure 2:**
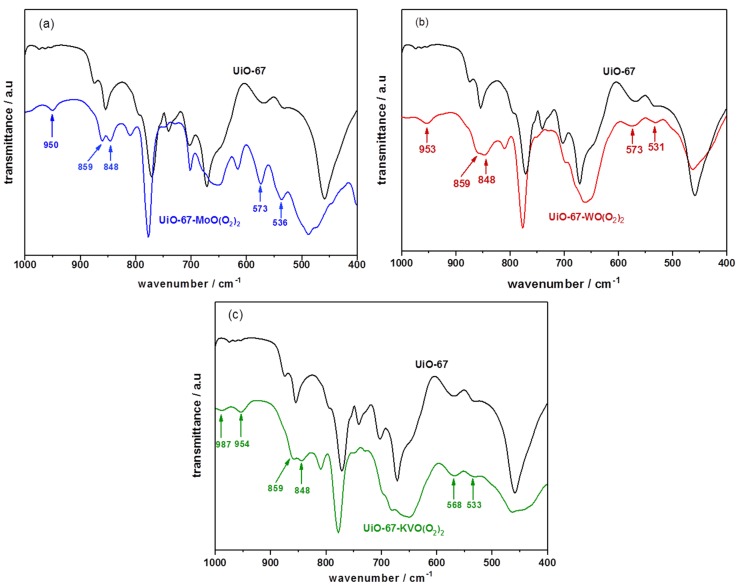
FTIR spectra of UiO-67, UiO-67-MoO(O_2_)_2_, UiO-67-WO(O_2_)_2_ and UiO-67-KVO(O_2_)_2_.

Figure 2 presents a summary of the FTIR spectra of the metal peroxide-modified UiO-67 in comparison with that of the pristine MOF. The band at 950 cm^−1^ is attributed to the Mo = O double bond stretching vibration in the octahedral molybdenum peroxide species in UiO-67-MoO(O_2_)_2_. The bands at 573 cm^−1^ and 536 cm^−1^ correspond to Mo-O with asymmetric and symmetric stretching vibration [40]. The band at 953 cm^−1^ is assigned to the W = O stretching vibration in the octahedral tungsten peroxide species in UiO-67-WO(O_2_)_2_, and the bands centered at 573 cm^−1^ and 531 cm^−1^ are attribute to the asymmetric and symmetric stretching vibration of W-O [41]. The bands at 987 cm^−1^ and 954 cm^−1^ are assigned to V = O double bond stretching vibrations in octahedral vanadium peroxide species in UiO-67-KVO(O_2_)_2_. The bands at 568 cm^−1^ and 533 cm^−1^ correspond to the vibration of V-O [44]. In all spectra of UiO-67-MO(O_2_)_2_ (M = Mo, W, V), shoulder bands at 859 cm^−1^ and 848 cm^−1^ correspond to the vibration of O-O [41,44], all pointing to the fact that peroxide species were present in the modified UiO-67 materials.

#### 3.1.4. SEM

**Figure 3:**
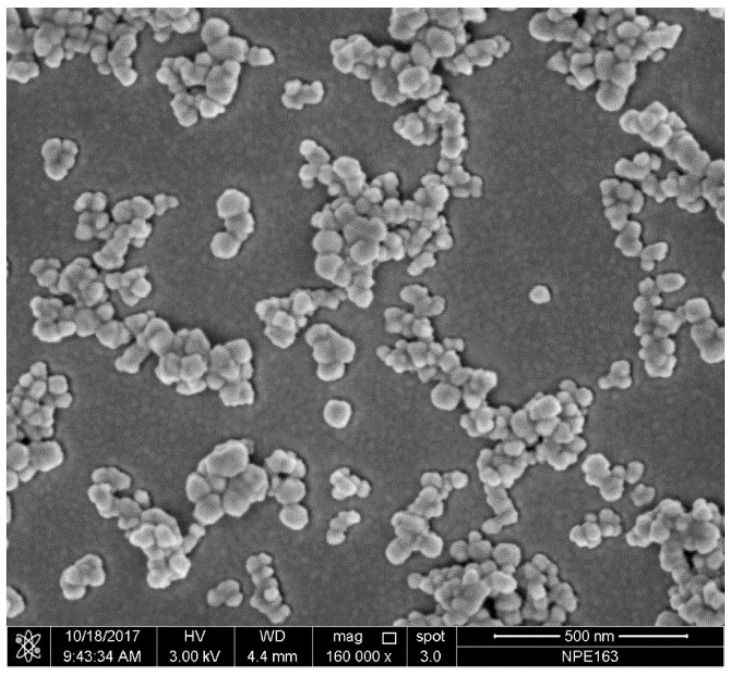
SEM of UiO-67-KVO(O_2_)_2_.

As shown in Figure 3, the SEM micrograph of UiO-67-KVO(O_2_)_2_ shows that the particle size was uniform at around 60 nm, but no obvious regular octahedron shape of UiO-67 material was observed. The reason for this might be due to the stirring during the synthesis, which has a negative effect on the growth of regular crystals.

#### 3.1.5. X-ray Photoelectron Spectroscopy

**Figure 4:**
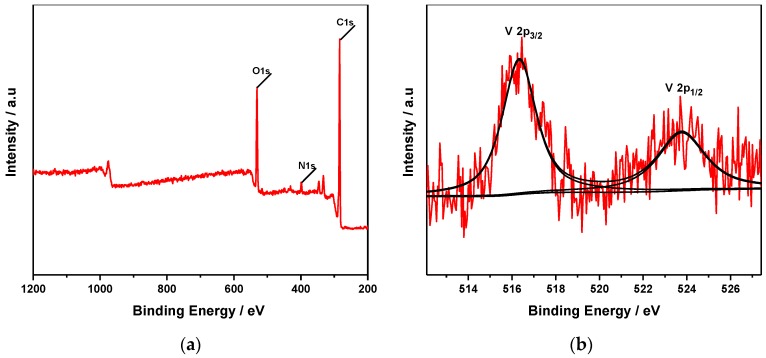
XPS spectra of UiO-67-KVO(O_2_)_2_ (**a**) and V 2p of UiO-67-KVO(O_2_)_2_ (**b**).

X-ray photoelectron spectroscopy (XPS) is an advanced analytical technique in microscopic analysis of electronic materials and components and is used for investigating the electronic characteristics of the species created on the surface, including the environment of electron, state of oxidation, and the binding energy of the core electron of the metal [45]. An XPS elemental survey scan of the surface of the UiO-67-KVO(O_2_)_2_ is shown in Figure 4. The peaks related to O 1 s, C 1 s, and N 1 s were observed in this spectrum (Figure 4a). In Figure 4b, UiO-67-KVO(O_2_)_2_ shows peaks at 523.7 eV and 516.3 eV, which correspond to V 2p_1/2_ and V 2p_3/2_ of V^5+^ [46].

### 3.2. Catalytic Performance

**Figure 5:**
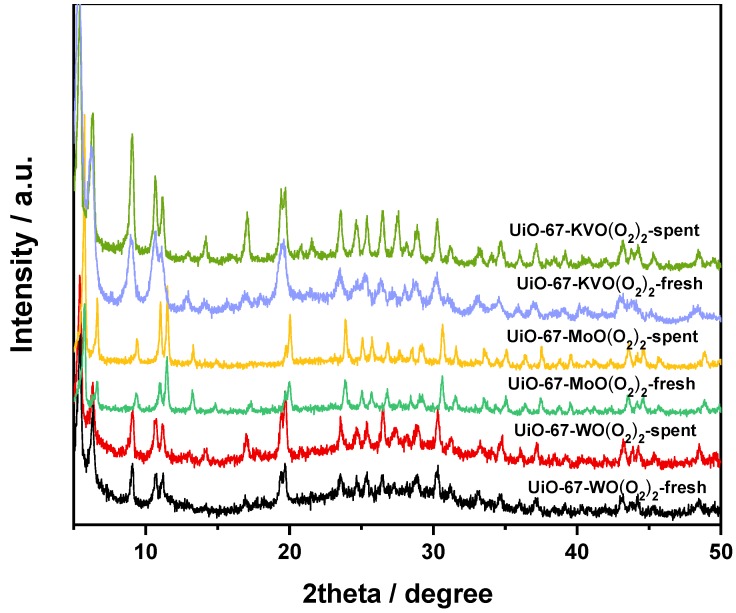
XRD patterns of UiO-67-MoO(O_2_)_2_, UiO-67-WO(O_2_)_2_, and UiO-67-KVO(O_2_)_2_ before and after reactions.

The catalytic activity of metal oxodiperoxo complex modified UiO-67 materials was investigated in the liquid phase oxidation of cyclohexane at 343 K with TBHP as the oxidant. The XRD patterns of the three metal oxodiperoxo complex modified UiO-67s before and after catalysis are shown in Figure 5. It is shown that the diffraction peaks were basically unchanged, indicating that the structural robustness of the material was fully retained during the reaction. The catalytic conversion and selectivity are shown in detail in Table 2. In the MOF modification process, the binding ability of different active metal components to the ligand is different, and it is difficult to ensure that the active metal M/MOF molar ratio is the same. Therefore, in the cyclohexane oxidation reaction, the amount of different catalysts was controlled based on the molar amount of the active metal. The molar ratio of cyclohexane to active metal (CH:M) was fixed to 266. Blank experiments were carried out to ensure the role of the active sites in the selective oxidation of cyclohexane. As summarized in Table 2, without adding any catalyst to the reaction mixture, negligible conversion of cyclohexane was observed; when UiO-67 was added to the reaction mixture, a low conversion of cyclohexane was observed, with CHHP as the major product. The formation of CHHP is mainly catalyzed by certain amounts of unsaturated zirconium sites on UiO-67. Compared to UiO-67-KVO(O_2_)_2_, UiO-67-MoO(O_2_)_2_ and UiO-67-WO(O_2_)_2_ catalyzed the cyclohexane oxidation with a very low cyclohexane conversion, and, similarly, CHHP was the main product, indicating only a minor contribution from the catalyst. On the other hand, it is clearly noticed that UiO-67-KVO(O_2_)_2_ was more active in the oxidation of cyclohexane, with a relatively higher cyclohexane conversion of 9.4% in 12 h. This observation was in agreement with the earlier report, which confirmed that the vanadium (V) oxodiperoxo complexes had the ability to transfer oxygen to aromatic hydrocarbons, acting as powerful epoxidizing and hydroxylating reagents.

**Figure 6:**
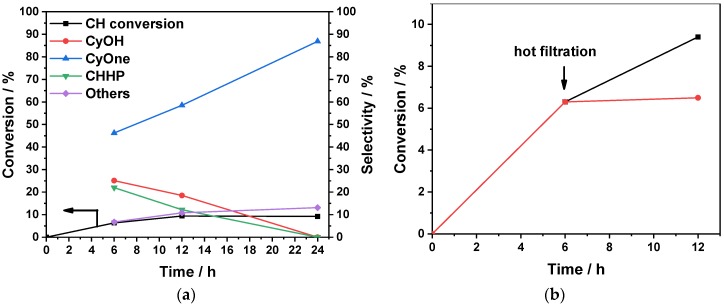
(**a**) Effect of reaction time on cyclohexane conversion and product selectivity catalyzed by UiO-67-KVO(O_2_)_2_. Reaction conditions: CH:M (molar ratio) = 266, T = 343 K; (**b**) Cyclohexane conversion (%) in the hot-filtration reaction over UiO-67-KVO(O_2_)_2_.

The trend in cyclohexane conversion and selectivity over time is shown in Figure 6a. The cyclohexane conversion increased to 6.3% in 6 h, slowly reached 9.4% conversion in 12 h, and remained almost constant afterward. However, the selectivities towards Cyone, CyOH, and CHHP were quite different over time. While increasing the reaction time, the selectivity toward Cyone had a constant increase from 46.2% at 6 h up to a maximum value of 86.9% at 24 h. The intermediate product, CHHP, decreased slowly, while the selectivity toward CyOH decreased over time as well, and no CyOH was observed after 24 h. While in a prolonged reaction timeframe, the selectivity to the by-product acids increased slowly. These results showed that a longer reaction time promoted the decomposition of the intermediate CHHP to afford the main product, CyOH and CyOne, whereas further prolongation of the reaction time led to an increased Cyone formation and decreased CyOH formation. The total selectivity toward the main product, KA oil, increased monotonically with time. Thus, the optimum reaction time was 24 h. To exclude the contribution of leached V species in the solution, a hot-filtration experiment was performed. After 6 h of reaction, the solid catalyst was removed from the reaction mixture by filtration, and the filtrate was recovered to continue the reaction under the same condition for an additional 6 h. Figure 6b indicates that the conversion of cyclohexane remained stable after the catalyst was filtered off, suggesting that the cyclohexane oxidation was terminated after removal of the solid catalyst (with the catalyst, the cyclohexane conversion reached 9.4% after 12 h).

**Figure 7:**
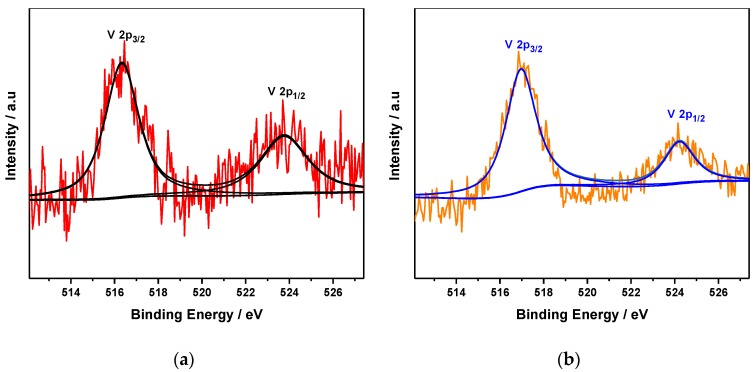
XPS spectra of V 2p of UiO-67-KVO(O_2_)_2_ before (**a**) and after (**b**) reactions.

The XPS spectra of UiO-67-KVO(O_2_)_2_ before and after reaction are illustrated in Figure 7. The V 2p_1/2_ and V 2p_3/2_ peak binding energy shifted from 523.7 eV and 516.3 eV (Figure 7a) to 524.4 eV and 517.0 eV (Figure 7b), respectively, with an 0.7 eV offset of both peaks. This is the characteristic of V^5+^ [47,48], confirming no obvious change in the valence state of V over the catalysis before and after reactions.

Among several metal oxodiperoxo complex modified UiO-67, UiO-67-KVO(O_2_)_2_ showed the best catalytic activity. In Table 3, a comparison between the reported vanadium-based silica, phosphorus, or mesoporous catalysts with UiO-67-KVO(O_2_)_2_ of the current study is summarized. All the catalytic reactions used TBHP as the oxidant, and UiO-67-KVO(O_2_)_2_ showed the second best selectivity toward KA oil (77%) with Cyone as the major product. V-Co-TUD-1, a cobalt promoted vanadium based mesoporous siliceous material, showed the best selectivity toward KA oil (87.9%) in a solvent free condition, and CyOH was the major product. The vanadium phosphorus oxide (VPO) and VPO-Co(0.10) (cobalt promoted VPO) catalysts showed similar selectivity toward KA oil, around 53%~55%, and the by-product for the VPO catalysts was mainly cyclohexene. However, cobalt doping significantly increased the overall activity for the oxidation of cyclohexane from 10% to 65%. Therefore, our future planning work includes the further tethering of Co^2+^ ions into UiO-67-KVO(O_2_)_2_ to investigate the effect toward the liquid phase oxidation of cyclohexane.

## 4. Conclusions

In the present study, characterization results confirmed that UiO-67-MoO(O_2_)_2_, UiO-67-WO(O_2_)_2_, and UiO-67-KVO(O_2_)_2_ was obtained, and the transition metal oxodiperoxo complexes (MoO(O_2_)_2_, WO(O_2_)_2_, and KVO(O_2_)_2_) were anchored on the framework by coordination. UiO-67-KVO(O_2_)_2_ showed the best catalytic performance in the liquid phase oxidation of cyclohexane with TBHP as the oxidant. The effect of reaction time in the reaction is critical. Cyclohexane conversion reached the highest value of 9.4% at 12 h, and further prolongation of reaction time led to an increased Cyone formation and a decreased CyOH formation. The selectivity towards the main product, KA oil, increased constantly over time. Hot-filtration experiment excluded the leaching of V species and its contribution to the catalytic reaction.

## Figures and Tables

**Table 1:** The specific surface area and mass fraction of active metal of modified metal-organic frameworks (MOFs).

MOFs	Metal Loading	Metal Loading	Metal/MOF	*S*_BET_
	wt%	mmol/g	Molar Ratio	m^2^/g
UiO-67	-	-	-	1562
UiO-67-MoO(O_2_)_2_	14.1 (Mo)	1.47 (Mo)	4.2 (Mo)	583
UiO-67-WO(O_2_)_2_	6.3 (W)	0.34 (W)	0.8 (W)	656
UiO-67-KVO(O_2_)_2_	2.7 (V)	0.53 (V)	1.2 (V)	305

**Table 2:** The result of cyclohexane oxidation catalyzed by metal peroxide modified UiO-67 ^a^.

Catalyst	Time/h	Conversion ^b^/%	Selectivity ^c^/%				
			CyOH	Cyone	KA oil	CHHP	Others ^d^
No catalyst	12	<1	-	-	-	-	-
UiO-67	12	1.3	13.4	35.8	49.2	50.8	-
UiO-67-MoO(O_2_)_2_	12	<1	-	33.2	33.2	66.8	-
UiO-67-WO(O_2_)_2_	12	1.6	16.2	23.2	39.4	60.6	-
UiO-67-KVO(O_2_)_2_	12	9.4	18.5	58.5	77.0	12.2	10.8
UiO-67-KVO(O_2_)_2_	24	9.2	-	86.9	86.9	-	13.1
UiO-67-KVO(O_2_)_2_	6	6.3	25.1	46.2	71.3	22.0	6.7
UiO-67-KVO(O_2_)_2_ after hot filtration ^e^	12	6.5	22.4	49.3	71.7	21.8	6.5
H_2_bpydc-KVO(O_2_)_2_	12	7.4	18.8	68.3	87.1	9.2	3.8

^a^ Reaction conditions: CH:M (molar ratio) = 266, CH:TBHP (molar ratio) = 1:1, T (temperature) = 343 K. ^b^ Conversion/% based on cyclohexane = total moles of products after reaction/moles of cyclohexane before reaction × 100. ^c^ Selectivity/% of product = moles of product after reaction/total moles of products. ^d^ Others are mainly organic acid (quantified based on the titration result with NaOH); no ester product was found based on the titration result. ^e^ Hot filtration: The reaction stopped after 6 h, the catalyst was filtered off, and the filtrate was left to continue to react under the same condition for another 6 h.

**Table 3:** Comparison of cyclohexane oxidation in MeCN solution by various vanadium based catalysts.

Catalyst	Time/h	Temp./°C	Conv. ^a^/%	Selectivity					Refs
				CyOH	Cyone	KA oil	CHHP	Others	
UiO-67-KVO(O_2_)_2_	12	70	9.4	18.5	58.5	77.0	13.1	10.8	This work
V-Co-TUD-1 ^c^	8	70	15.9	62	25.9	87.9	2.4	9.7	[21]
VPO	8	90	10	33	22	55	-	45 ^b^	[49]
VPO-Co(0.10)	8	90	65	20	33	53	-	47 ^b^	[49]

^a^ TBHP as the oxidant, ^b^ Cyclohexene, ^c^ Solvent free.

## Supplementary Materials

The following are available online at https://www.mdpi.com/1996-1944/13/4/829/s1, Figure S1: FT-IR spectra of H_2_bpydc, H_2_bpydc-KVO(O_2_)_2_ and H_2_bpydc-WO(O_2_)_2_, Figure S2: XRD patterns of UiO-67-MoO(O_2_)_2_ and UiO-67-KVO(O_2_)_2_ in different drying temperature, Table S1: The ICP and elemental analysis results of H_2_bpydc-KVO(O_2_)_2_ and H_2_bpydc-WO(O_2_)_2_.
