# Supplementary material for: Transition Metal Oxodiperoxo Complex Modified Metal-Organic Frameworks as Catalysts for the Selective Oxidation of Cyclohexane
Source: Materials (Basel). 2020 Feb 12;13(4):829. doi: 10.3390/ma13040829 (PMC7078608; doi:10.3390/ma13040829)
Supplement: Supplementary file 1 [file materials-13-00829-s001.pdf]

materials

Article

# Transition Metal Oxodiperoxo Complex Modified Metal-Organic Frameworks as Catalysts for the Selective Oxidation of Cyclohexane

**Yuechao Hong[1], Jie Peng[1], Zhichao Sun[1], Zhiquan Yu[1], Anjie Wang[1], Yao Wang[1], Ying-Ya Liu[1,*] Fen Xu[2] and Li-Xian Sun[2]**

1 State Key Laboratory of Fine Chemicals, School of Chemical Engineering, Dalian University of Technology, Dalian 116024, China; h15541172820@163.com (Y.H.); peng.jie.p4@dc.tohoku.ac.jp (J.P.); sunzhichao@dlut.edu.cn (Z.S.); yuzhiquan@dlut.edu.cn (Z.Y.); ajwang@dlut.edu.cn (A.W.); wangyao@dlut.edu.cn (Y.W.)

2 Guangxi Key Laboratory of Information Materials and Guangxi Collaborative Innovation Center of Structure and Property for New Energy and Materials, School of Material Science & Engineering, Guilin University of Electronic Technology, Guilin 541004, China; xufen@guet.edu.cn (F.X.); sunlx@guet.edu.cn (L.-X.S.)

* Correspondence: yingya.liu@dlut.edu.cn

Received: 28 December 2019; Accepted: 10 February 2020; Published: February 2020

**Table S1.** The ICP and elemental analysis results of $H_2bpydc\text{-}KVO(O_2)_2$ and $H_2bpydc\text{-}WO(O_2)_2$.

| Metalated Ligand | Formula | | Metal Loading wt% | C (wt%) | H (wt%) | N (wt%) |
|---|---|---|---|---|---|---|
| $H_2bpydc\text{-}KVO(O_2)_2$ | $C_{12}N_2O_4H_8(KVO_5)_{0.35}(H_2O)_{0.7}$ | Exp. | 5.5(V) | 42.6 | 2.5 | 8.3 |
| | | Theo. | 5.6(V) | 45.5 | 2.9 | 8.8 |
| $H_2bpydc\text{-}WO(O_2)_2$ | $C_{12}N_2O_4H_8(WO_5)_{0.31}$ | Exp. | 17.6(W) | 47.4 | 2.8 | 9.2 |
| | | Theo. | 17.5(W) | 44.2 | 2.5 | 8.6 |

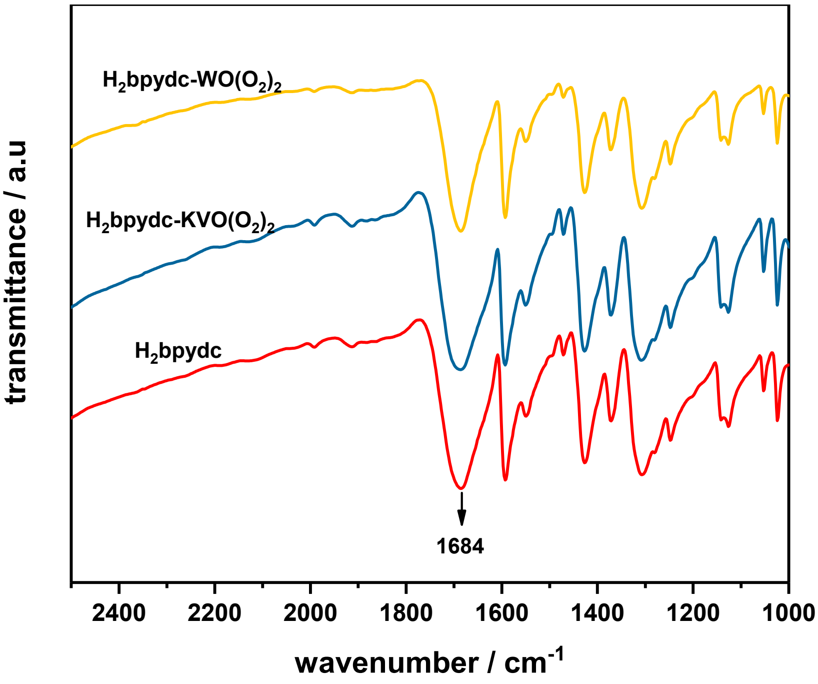

**Figure S1 .** FT-IR spectra of $H_2bpydc$, $H_2bpydc$-$KVO(O_2)_2$ and $H_2bpydc$-$WO(O_2)_2$.

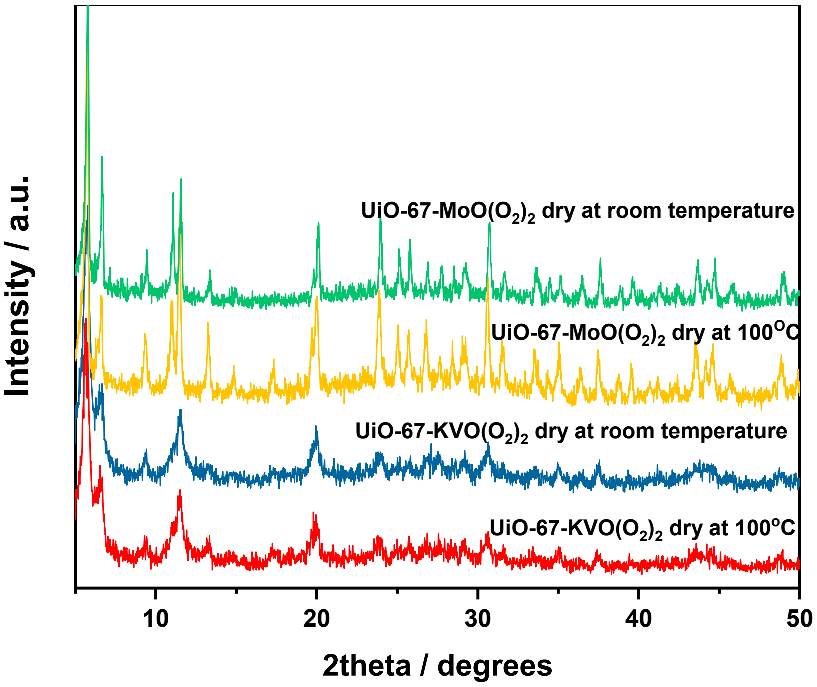


**Figure S2.** XRD patterns of UiO-67-$MoO(O_2)_2$ and UiO-67-$KVO(O_2)_2$ in different drying temperature.

© 2020 by the authors. Submitted for possible open access publication under the terms and conditions of the Creative Commons Attribution (CC BY) license (http://creativecommons.org/licenses/by/4.0/).
